# Large Scalp Defect Reconstruction With Tissue Expansion, Orticochea Flap, and Acellular Dermal Matrix for Soft Tissue Augmentation: A Case Report

**DOI:** 10.7759/cureus.27723

**Published:** 2022-08-05

**Authors:** Tru Tran, Jeanne Le, Jacqueline Royce

**Affiliations:** 1 College of Osteopathic Medicine, Lake Erie College of Osteopathic Medicine, Bradenton, USA; 2 Plastic and Reconstructive Surgery, Lake Erie College of Osteopathic Medicine, Bradenton, USA

**Keywords:** scalp reconstruction, large scalp defect, acellular dermal matrix, orticochea flap, tissue expansion

## Abstract

Reconstruction of a large scalp defect following oncologic surgical resection is a challenging task. The defect size, location, and elasticity of the soft tissue overlying the calvarium are important factors to be considered when exploring available reconstructive options. When primary closure is not feasible with a large defect, a skin flap or graft is utilized. Skin flap is advantageous as it produces a similar color and texture as the surrounding areas, thus being the favorable method. Wounds involving exposed bone, tendon, and cartilage cannot support grafts due to poor vascularity and thus require a skin flap. One of the multi-flap closure modalities, the Orticochea flap, is an excellent choice for scalp reconstruction on large defects greater than 50 cm^2^. We present an interesting case of a patient with a large scalp defect following Mohs surgery of basal cell carcinoma (BCC) and squamous cell carcinoma (SCC) that was successfully reconstructed with tissue expansion utilizing Orticochea flap, with the addition of an acellular dermal matrix as an adjunct in such hostile scalp reconstruction.

## Introduction

We present the case of a 53-year-old female who presented with basal cell carcinoma (BCC) of the scalp and required scalp construction following Mohs surgery. Scalp basal cell carcinoma is one of the rarer types of basal cell carcinomas.

## Case presentation

**Figure 1:**
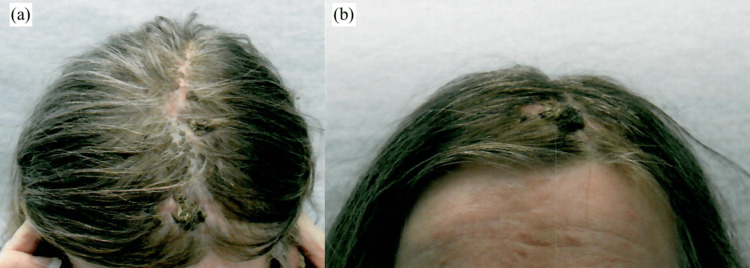
Preoperative cancerous lesions: (a) superior view and (b) anterior view.

A 53-year-old Caucasian female with a past medical history of numerous actinic keratoses, basal cell carcinoma (BCC), and squamous cell carcinoma (SCC) throughout the body presented with a history of extensive crusting lesions on the superior central scalp measuring 12 × 6.5 cm (Figure 1). Histopathological examination of the punch biopsy specimen revealed a moderately differentiated SCC measuring 4.6 × 3.4 cm on the left superior central forehead and right superior medial forehead and nodular BCC measuring 3.6 × 3.5 cm located on the left central frontal scalp. Considering the moderate risk of metastasis, lesion size, and moderate differentiation, computed tomography (CT) with contrast of the head and neck was ordered for the evaluation of draining nodal basins.

**Figure 2:**
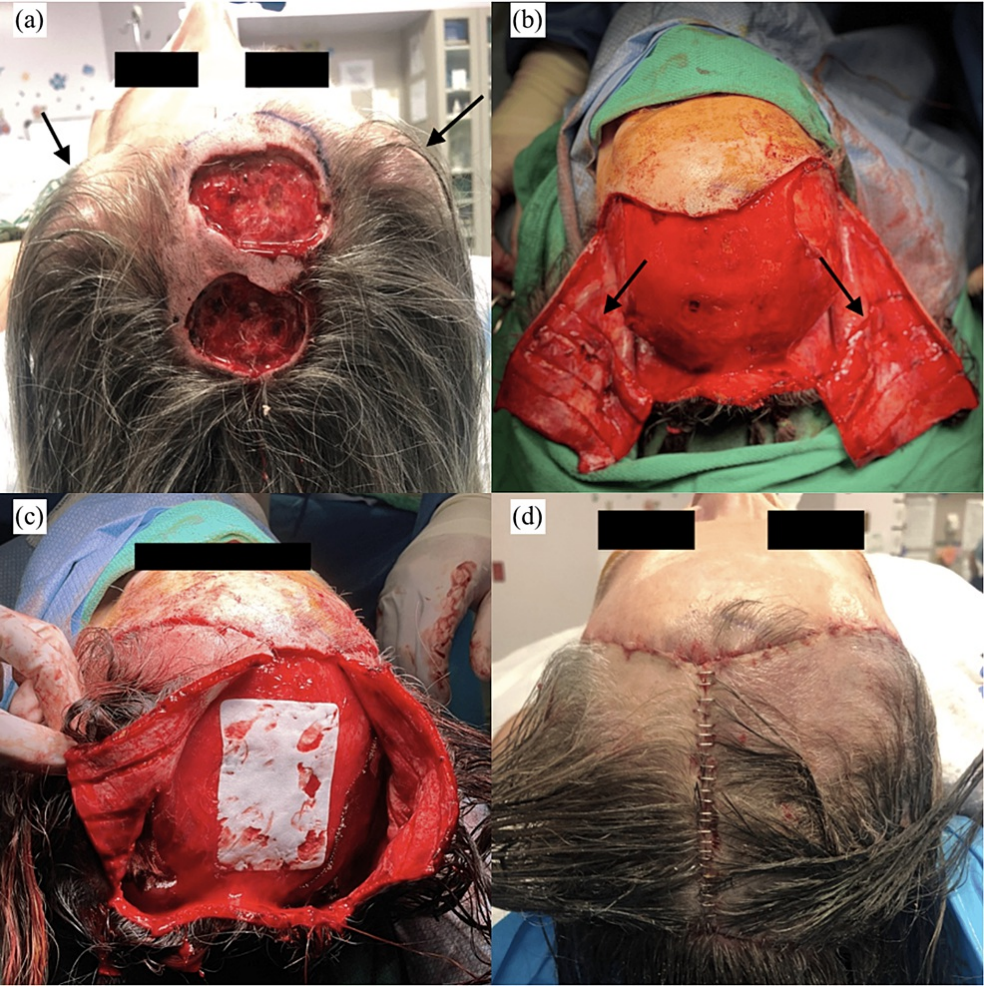
(a) Intraoperative scalp defect following the resection of cancerous lesions (arrows: a smooth crescent-shaped tissue expander on the right parietal scalp and two smooth crescent-shaped tissue expanders on the left parietal scalp were placed under the epicranial aponeurosis and expanded with 8-30 mL of saline weekly). (b) Orticochea flap and scoring of the galea aponeurosis (arrows). (c) Placement of an acellular dermal matrix at the closure line. (d) Immediate post-reconstruction of scalp defect.

The patient elected to undergo tissue expansion in preparation for wound defect closure following Mohs surgery. A 200-mL smooth crescent-shaped tissue expander (Mentor, Santa Barbara, CA, USA) on the right parietal scalp and two 100-mL Mentor smooth crescent-shaped tissue expanders on the left parietal scalp were placed under the epicranial aponeurosis and expanded with 8-30 mL of saline weekly (Figure 2a). Following full inflation of the tissue expanders in two months, excision of primary cancer by Mohs surgery was performed by the dermatologist, which resulted in a large full-thickness skin defect measuring 12 × 6.5 cm (Figure 2a). The tissue expanders were removed, and the defect was reconstructed using Orticochea flaps without the need for skin grafts. Prior to closing, a MatriDerm graft measuring 5.2 × 7.4 cm was placed centrally for improved healing and circulation (Figures 2c, 2d).

During the one-month postoperative follow-up period, the patient’s scalp healed well without any evidence of wound breakdown, infection, or necrosis.

## Discussion

Tissue expanders allow for primary closure in procedures involving large skin defects. The expansion of the native tissue maintains vascularity and provides adequate skin needed for proper tension-free closure [1,2]. In this case, the gradual expansion of the scalp tissue preceded the wide excision of cancerous lesions. Other authors have achieved rapid intraoperative tissue expansion using a Foley catheter, a readily available and economical means of tissue expansion that reduces the risk of infection and eliminates the need for multiple visits for chronic expansion [3]. However, this case included a significantly large surface defect that limits the efficacy of catheter expansion and favors chronic expansion.

**Figure 3:**
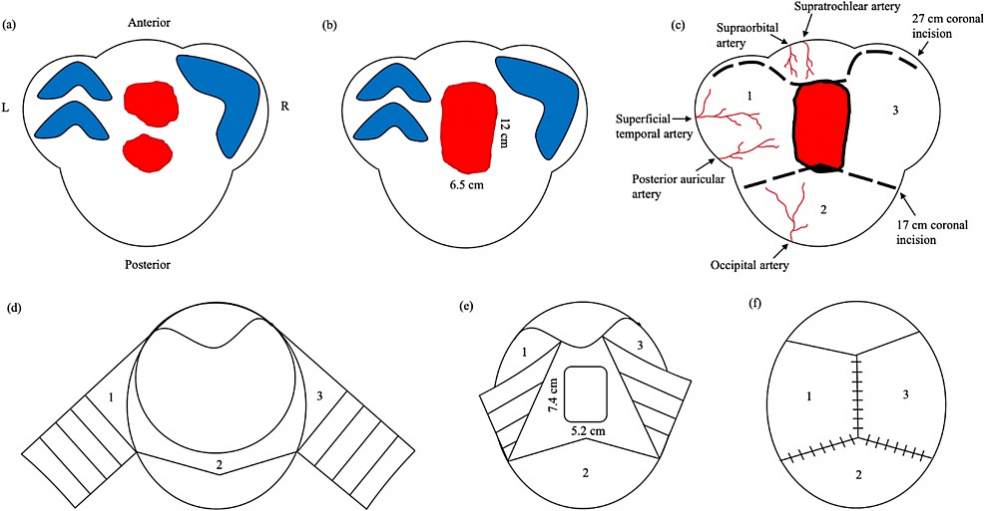
(a) Two large scalp defects following Mohs surgery. (b) Singular 12 × 6.5 cm defect created. (c) Incision outlines and arterial blood supply of the scalp. (d) Constructing advancement flaps with scoring of the galea aponeurosis. (e) Placement of the MatriDerm graft at the closure line. (f) Tension-free primary closure with sutures and staples.

The scalp reconstruction began with two large defects post-Mohs surgery by the dermatologist (Figure 3a). The skin between the two defects was excised to create a single defect (Figure 3b). The scalp is supplied by the branches of the internal and external carotid arteries that have extensive collateralization and include the supraorbital artery, superficial temporal artery, and occipital artery (Figure 3c). Local flaps for scalp reconstruction should incorporate at least one of the major scalp arteries. Scalp reconstruction of the frontal and occipital regions by Orticochea flap is a relatively newer technique that involves three flaps instead of the standard four flaps, thus allowing for a greater surface area available for closure while maintaining vascularity in each flap [4]. The two advancement flaps measured 10 cm wide each, forming the left and right parietal flaps that measured a total of 270 cm^2^ throughout. The galea aponeurotica was scored in a horizontal fashion, perpendicular to the tension line, to enhance elasticity and extend the flaps for closure (Figures 2b, 3d). The amount of skin dissected for reconstruction was adequate for tension-free primary closure and avoided the need for skin grafting (Figures 3e, 3f). The Orticochea flap is preferred because the use of the existing hair-bearing scalp in reconstruction avoids the need for hair transplantation with skin grafting.

Large skin defects created by wide local excision expose the cranial bone, leading to the disruption of blood supply and slowed healing. Dermal substitute is an acellular bovine tissue matrix made of collagen fibrils and elastin upon which dermal regeneration can occur for improved healing, hemostasis, and elasticity of the skin [5]. Commonly used as an adjunct with skin grafts, dermal substitutes (MatriDerm®, MedSkin Solutions Dr. Suwelack AG, Billerbeck, Germany) promote fibroblast and keratinocyte migration for accelerated wound closure, re-epithelialization, and wound closure [6]. The dermal substitute was prepared and applied according to the manufacturer’s guidelines.

## Conclusions

Successful reconstruction of the scalp requires a comprehensive preoperative plan and meticulous intraoperative execution. The employment of tissue expanders and Orticochea flap provided additional scalp tissue for advancement flap closure. Our case demonstrates the use of both techniques as an effective method for scalp reconstruction by utilizing existing tissue to repair large scalp defects without extensive anesthesia, prolonged operative times, and potential surgical complications.
